# Dysregulation of SOCS-Mediated Negative Feedback of Cytokine Signaling in Carcinogenesis and Its Significance in Cancer Treatment

**DOI:** 10.3389/fimmu.2017.00070

**Published:** 2017-02-08

**Authors:** Mengmeng Jiang, Wen-wen Zhang, Pengpeng Liu, Wenwen Yu, Ting Liu, Jinpu Yu

**Affiliations:** ^1^Department of Immunology, Key Laboratory of Cancer Immunology and Biotherapy, National Clinical Research Center of Cancer, Tianjin Medical University Cancer Institute and Hospital, Tianjin, China; ^2^Key Laboratory of Cancer Prevention and Therapy, Clinical Research Center for Cancer, Tianjin Medical University Cancer Institute and Hospital, Tianjin, China; ^3^Cancer Molecular Diagnostic Center, Key Laboratory of Cancer Prevention and Therapy, National Clinical Research Center of Cancer, Tianjin Medical University Cancer Institute and Hospital, Tianjin, China

**Keywords:** SOCS1, SOCS3, dysregulation, carcinogenesis, immune cells, target therapy

## Abstract

Suppressor of cytokine signaling (SOCS) proteins are major negative feedback regulators of cytokine signaling mediated by the Janus kinase (JAK)-signal transducer and activator of transcription signaling pathway. In particular, SOCS1 and SOCS3 are strong inhibitors of JAKs and can play pivotal roles in the development and progression of cancers. The abnormal expression of SOCS1 and SOCS3 in cancer cells is associated with the dysregulation of cell growth, migration, and death induced by multiple cytokines and hormones in human carcinomas. In addition, the mechanisms involved in SOCS1- and SOCS3-regulated abnormal development and activation of immune cells in carcinogenesis, including T cells, macrophages, dendritic cells, and myeloid-derived suppressor cells, are still unclear. Therefore, this study aims to further discuss the molecules and signal pathways regulating the expression and function of SOCS1 and SOCS3 in various types of cancers and elucidate the feasibility and efficiency of SOCS-based target therapeutic strategy in anticancer treatment.

## Introduction

The occurrence and progression of cancer are closely linked to the microenvironment of tumor. In tumor microenvironment, many inflammatory cytokines, such as interleukin (IL)-6 (1), granulocyte colony-stimulating factor (G-CSF) (2), interferon (IFN)-α (3), IFN-γ (4), and leptin (5), contribute to tumorigenesis by promoting tumor growth and invasion. Cytokines regulate the proliferation, apoptosis, and migration of tumor cells by binding to cell-surface receptors and activating intracellular signal transduction cascades. Janus kinase (JAK)-signal transducer and activator of transcription (JAK/STAT) signaling pathways are most frequently activated upon cytokine stimulation. However, the activation of JAK/STAT signaling pathways in normal cells is short and quick because of the rapid triggering of varied negative feedback loops, which efficiently block the STAT signaling pathways and the activities of downstream functional proteins (1). Such biological process is disrupted by dysfunctional negative feedback loops, which induce the constitutive activation of STAT signaling pathway, oncogenic transformation, tumor cell invasion, and metastasis (6). At least three kinds of inhibitors, namely, protein tyrosine phosphatases (such as SHP-1 and CD45), protein inhibitors of activated STATs, and suppressor of cytokine signaling (SOCS) proteins, contribute to the negative regulation of cytokine signaling. Among these inhibitors, the SOCS family includes the most frequent and important inducible negative regulators upon cytokine stimulation.

**Figure 1:**
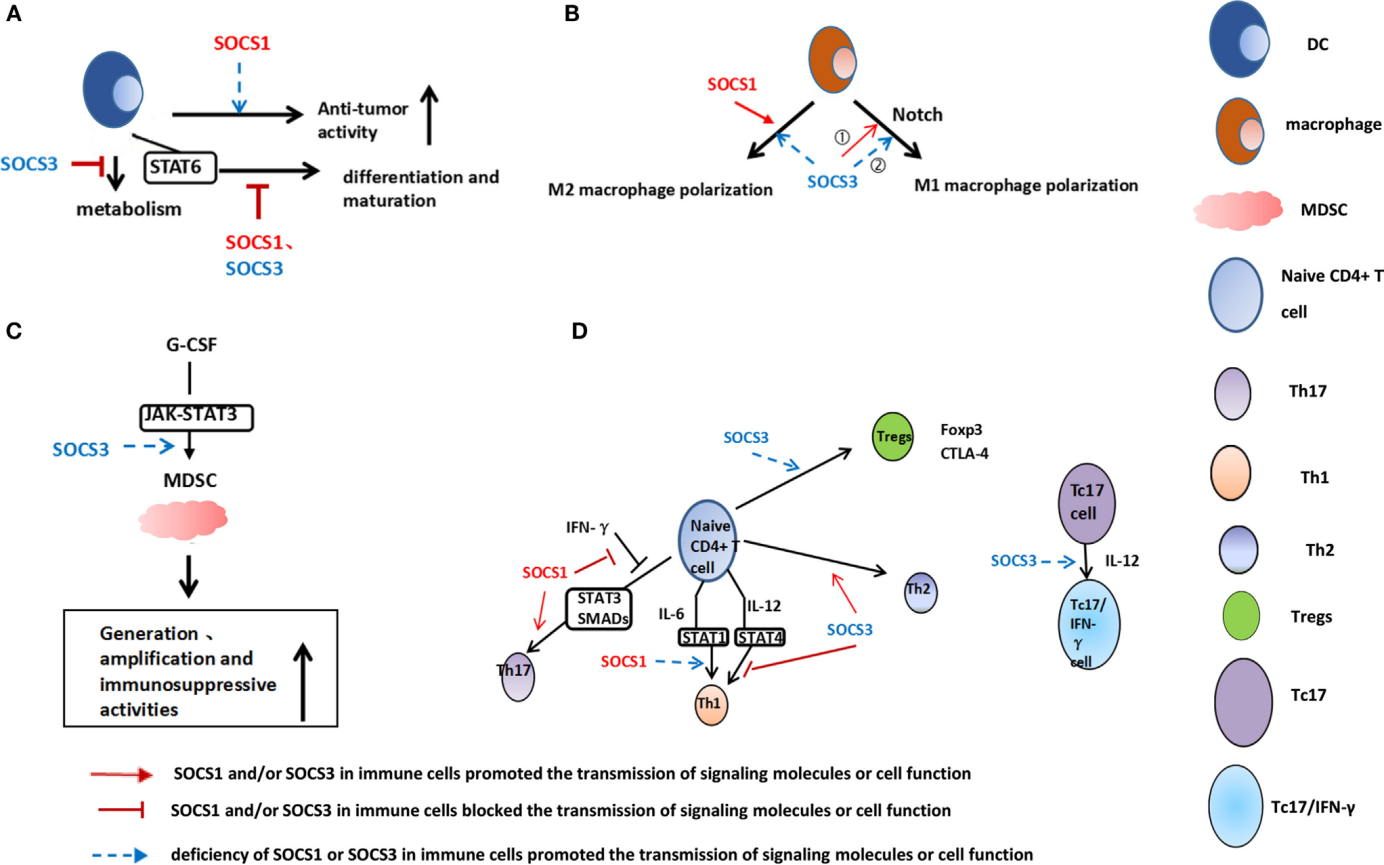
**Role of suppressor of cytokine signaling (SOCS) 1 and SOCS3 in dendritic cells (DCs) (A), macrophages (B), myeloid-derived suppressor cells (MDSCs) (C), and T-cells (D)**. **(A)** SOCS1-silenced DCs induce an enhanced antigen-specific CTL response and antitumor activity; STAT6 signaling is inhibited by SOCS1, and SOCS3 may be required for DCs maturation. SOCS3 promotes indoleamine 2,3-dioxygenase degradation and enhances DCs antitumor effects. **(B)** SOCS3 could regulate macrophage polarization. (1) SOCS3 deficiency in macrophages prevents M1 activation and promotes anti-inflammatory responses. Therefore, SOCS3 is necessary for M1 macrophage polarization in inflammatory diseases. (2) However, SOCS3 deficiency in macrophages prevents cancer metastasis and suppresses tumor growth, which is related to elevated M1 cytokines levels. SOCS1 promotes M2 phenotype. **(C)** Genetic deletion of SOCS3 in myeloid cells significantly elevates the levels of MDSCs and increases the immunosuppressive activities. **(D)** SOCS1-deficient naive CD4^+^ T cells are predominantly differentiated into Th1 and poorly into Th17. SOCS1 is necessary for Th17 differentiation by suppressing the antagonistic effect of interferon (IFN)-γ on both STAT3 and SMAD. SOCS3 promotes Th2 and inhibits Th1 differentiation through inhibition of interleukin (IL)-12-mediated STAT4 activation. Tregs are deficient in SOCS3 protein expression. IL-12 permits the conversion of mouse IL-17-producing CD8^+^ T (Tc17) cells to IL-17/IFN-γ-double producing CD8^+^ T cells. Such conversion is caused by the prohibitive epigenetic modifications of SOCS3 gene promoters.

To date, over 900 publications regarding the relationship between the SOCS family and cancer exist (7). The abnormal expression of SOCSs regulates tumor development in various tumor cells. Among them, SOCS1 and SOCS3 are the critical molecules in the development of cancers (Table 1) (8–10). The abnormal expression of SOCSs has been discovered not only in cancer cells but also in immune cells in the tumor microenvironment. SOCS1 and SOCS3 are important regulators of tumor-infiltrated T cell, dendritic cells (DCs), myeloid-derived suppressor cells (MDSCs), and macrophages (Figure 1) (11–13). Therefore, the present study focused on the similarity and disparity of the expression, distribution, function, and regulation of SOCS1 and SOCS3 between tumor cells and immune cells in the tumor microenvironment. Consequently, the feasibility and efficiency of SOCS-based target therapeutic strategy in anticancer treatment were elucidated.

**Table 1:** **Suppressor of cytokine signaling (SOCS) 1 and SOCS3 are critical molecules in the development of cancers**.

Genes	Cell types	Data sources	Expression	Function	Regulated target genes	Reference
SOCS1	Non-small-cell lung cancer cell	Cell lines	Upregulation	Suppress proliferation	STAT3, Janus kinase (JAK) 1, JAK2, and FAK	(33)
		Mouse xenograft model				
		Cell lines	Downregulation	Enhance cell growth, viability, invasion, and migration	STAT3	(91)

	Prostate cancer cell	Cell lines Human samples	Downregulation	Cause a potent growth stimulation of cell lines	Cyclins D1 and E, cyclin-dependent kinases2 (CDK2), CDK4	(34)

	Colorectal tumor cell	Human samples Cell lines Mouse xenograft model	Upregulation Upregulation	The expression level was negatively related with tumor stages Prevent the EMT and metastases	E-cadherin, ZEB1, and fibronectin-1	(35)

	Breast cancer cell	Human samples Cell lines Human samples Cell lines	Upregulation Downregulation	Better clinical outcomes May elicit increased epithelial proliferation and/or survival in response to cytokines		(37) (73)

	Chronic myeloid leukemia cell	Human samples	Upregulation	A shorter progression-free survival time Confer resistance against cytokine therapy	Interferon (IFN)-α	(41)

	Lymphoma cell	Cell lines Human samples	Downregulation	Promote cell proliferation	JAK2 and STAT5	(80)

	Dendritic cells (DCs)	Mice model	Downregulation	Induce an enhanced antitumor inflammation and suppress tumor development Induce stronger Th1-type responses Induce maturation of DCs	IL-12 IFN-γ/STAT1 STAT1	(51) (52) (53)

	T cells	Mice model	Upregulation	Inhibit Th1 differentiation Is necessary for Th17 differentiation	STAT1 STAT3 and SMADs	(67) (69)

SOCS3	Breast cancer cell	Cell lines Mouse xenograft model	Downregulation Upregulation	Upregulate inflammatory cytokine IL-6 Inhibit tumor growth and reduce circulating tumor cells	IL-6/STAT3/NF-κB	(47)

	Prostate cancer cell	Cell lines	Upregulation	Inhibit androgen-mediated proliferation and secretion	CDK2, CDK4, cyclins E, and D1	(44)

	Melanoma cell	Cell lines	Upregulation	Influence the responsiveness of melanoma cells to IFN-a and IFN-γ	STAT1, ISG-15, OAS1, and IRF1	(49)

	Malignant pleural mesothelioma cell	Cell lines Pleural xenograft model	Upregulation	Induce apoptosis and partial G0/G1 arrest and inhibit tumor growth	JAK/STAT3, ERK, FAK, and p53	(45)

	Cholangiocarcinoma cell	Cell lines Human samples	Downregulation Downregulation	Resistance to apoptosis	IL-6/STAT3 IL-6/STAT3	(74) (75)

	Head and neck squamous cell carcinoma cell	Human samples Cell lines	Downregulation Upregulation	Cause growth inhibition and apoptosis	STAT3, Bcl-2, Bcl-xL	(8)

	Hepatocellular carcinoma cell	Cell lines	Downregulation	Promote cell growth and migration	IL-6/JAK/STAT3 and FAK	(9)

	Lung cancer cell	Cell Lines Human samples Cell Lines	Downregulation Upregulation	Induce apoptosis and growth suppression	STAT3	(76)

	DCs	Mice model	Upregulation	Degradation of IDO	IDO	(55)

	Macrophage	Rat model	Downregulation	Prevent M1 activation, promote anti-inflammatory responses Have a striking bias toward M2-like population Promote M1 macrophage polarization	STAT3 STAT3	(13, 62) (63)
			Downregulation	Prolong survival in a glioma model and suppress glioma tumor growth Prevent cancer metastasis	STAT3 MCP2/CCL8	(64, 65) (65) (61)

	MDSCs	Mice model	Downregulation	Negatively regulate MDSCs development, promote tumor growth, suppress antigen-specific T-cell responses	STAT3	(66)

	T cells	Mice model	Upregulation Downregulation	Is necessary for Th2 differentiation Increase Tregs proliferation	IL-12/STAT4 CTLA-4	(68) (71)

## SOCS Protein Family

The SOCS protein family consists of eight classical members, including SOCS1–7 and cytokine-induced Src homology (SH2)-containing protein (CIS) (7). Members of the SOCS family present similar structures, such as a conserved SH2 domain, extended SH2 domain, a SOCS box in the C-terminal, and an N-terminal region of variable length. In addition, SOCS1 and SOCS3 possess a kinase inhibitory region (KIR) that serves as a pseudosubstrate for JAKs, thereby blocking the JAK function. According to orthologous features, SOCS proteins are divided into three subgroups: CIS and SOCS1–3, SOCS4/5, and SOCS6/7 (14, 15). CIS and SOCS1–3 group is strictly associated with the control of cytokine signaling (4, 16), whereas SOCS4–7 homologs regulate the growth factor-induced receptor tyrosine kinase signaling (17, 18).

Among the SOCS subfamily, SOCS1–3 are well characterized and the most potent negative regulators of proinflammatory cytokine signaling through triggering a feedback loop on the JAK/STAT pathway (19, 20). Upon activation of JAKs, SOCS-mediated negative regulation loop is initiated. First, the conserved SOCS box in the C-terminus recruits Elongin B/C, Cullin 2, and Ring-box 2 to form an ubiquitin E3 ligase complex, which promotes the degradation of SH2-binding proteins by proteasome (21). Afterward, the SH2 domain competitively combines with the phosphorylated tyrosine sites of the cytokine receptors and prevents STAT activation (1). Moreover, the KIR structures in SOCS1 and SOCS3 interact with the phosphotyrosine sites on JAK kinases to hinder the activation of STAT proteins (22, 23). Both SOCS1 and SOCS3 directly inhibit JAK1, JAK2, and TYK2 (24–26). In addition, SOCS1 and SOCS2 display critical roles in regulating STAT3 and STAT5 activation (27, 28), and SOCS3 can regulate other STAT pathways, such as STAT1 (29) and STAT4 (30).

## SOCS and tumors

### SOCS1 in Tumor

Suppressor of cytokine signaling 1 expression is affected by various cytokines and hormones. SOCS1 displays a potent antiproliferative effect on tumor cells and is dependent on the inhibition of STAT3 and on other signaling proteins (31). In non-small cell lung cancer cells, SOCS-1 inhibits the activity of FAK-dependent signaling pathway, except for JAK/STAT signaling, by suppressing the phosphorylation of FAK tyrosine (32, 33) and promoting polyubiquitination and the degradation of FAK in a SOCS box-dependent manner (21). In prostate cancer, SOCS1 expression decreases after androgen-deprivation treatments and is elevated in recurrent patients to inhibit cell proliferation. SOCS1 also exerts a growth-inhibitory function through downregulation of cyclin D1, cyclin-dependent kinases2 (CDK2), and CDK4, in prostate cancer (34). Furthermore, SOCS1 inhibits the invasion and migration of colorectal tumors by preventing the epithelial–mesenchymal transition and promoting the mesenchymal–epithelial transition by increasing E-cadherin and decreasing ZEB1 observed in cell lines and mouse xenograft models (35). SOCS1 expression exhibits a correlation with the clinical stages of colorectal tumors. Tumors at stages II–IV express lower levels of SOCS1 mRNA than those of tumors at stage I; additionally, SOCS1 protein is highly expressed in all of the well-differentiated adenocarcinomas (35). Thus, SOCS1 can be regarded as a molecular marker of the disease progression of colorectal tumors. SOCS1 expression in breast tumor tissues and cell lines might be caused by proinflammatory cytokine, growth hormone, and prolactin in the tumor microenvironment (36, 37), which result in the loss of sensitivity to subsequent prolactin stimulus (38). Breast cancer patients with positive expression of SOCS1 protein exhibit a decreased risk of detectable circulating tumor cells in the peripheral blood. Therefore, SOCS1 is critical for the successful control of tumor dissemination (39). High expression level of SOCS1 mRNA is associated with early tumor stages and improved clinical outcomes in breast cancer (40). SOCS1 expression in chronic myeloid leukemia correlates with poor cytogenetic response to IFN-α and short progression-free survival time (41). Evidence indicated that SOCS1 downregulates the biological functions of IFN (42, 43). Hence, aberrant SOCS1 expression might induce the resistance against IFN-α therapy and cause poor prognosis, which implied that SOCS1 might be a negative prognostic biomarker in the chronic myeloid leukemia treated with IFN-α.

Taken together, SOCS1 shows an antitumor effect in most of tumors through inhibiting tumor proliferation, attenuating tumor invasion, and reducing the sensitivity of tumor cells to cytokines or hormones. The expression of SOCS1 is associated with clinical stages of tumor. In tumors at early stage, elevated SOCS1 is frequently observed compared with those tumors at advanced stage, which could be regarded as a molecular marker of cancer progression. However, the concrete molecular mechanisms regulating the expression of SOCS1 in different stages of tumors have not been fully elucidated.

### SOCS3 in Tumor

Suppressor of cytokine signaling 3 can act either as an oncogene or a tumor suppressor, depending on cellular context. SOCS3 is frequently silenced in cancer, thereby leading to a growth advantage for cancer cells. Similar to SOCS1, SOCS3 significantly diminishes the cell cycle regulatory proteins CDK2 and CDK4, cyclin E, and cyclin D1 in prostate tumor (44). The overexpression of SOCS3 induces apoptosis and partial G0/G1 arrest by inhibiting JAK1, ERK, and FAK signaling pathways in malignant pleural mesothelioma EHMES-1 and H226 cell lines. Furthermore, SOCS3 exhibits antitumor activity in a mesothelioma xenograft model, such as reducing tumor volume and tumor nodule weight and inducing tumor apoptosis (45).

Suppressor of cytokine signaling 3 can potentially hinder the receptor recruitment of the SH2 domain-containing tyrosine phosphatase to Tyr759 of gp130, which results in inhibition of ERK activation (9, 46). Methylation silencing of SOCS3 increases the IL-6/JAK/STAT3 and FAK phosphorylation in human hepatocellular carcinoma (9). The restoration of SOCS3 decreases STAT3 and FAK phosphorylation and FAK protein level, thereby inhibiting cell migration and growth (9). SOCS3 can also negatively control the STAT3/NF-κB signaling pathway in a triple-negative breast cancer model. Downregulation of SOCS3 correlates with increased level of IL-6 (47). The molecular and mutational analyses of breast cancers revealed that inactivation of tumor suppressors, p53, and PTEN is accompanied with downregulation of SOCS3, which is forcefully associated with the development and progression of triple-negative breast cancer (47). Additionally, the knockdown of p53 and PTEN in MCF10 cells promotes the downregulation of SOCS3 at translational/posttranslational level; such action is attributed to the proteasomal degradation after the activation of proinflammatory IL-6/STAT3/NF-κB signaling pathway rather than DNA methylation in colorectal and hepatocellular carcinomas (9, 48). This study provided a new understanding regarding the mechanism of SOCS3 silencing. SOCS3 can be also detected in breast cancer tissue samples because the number of analyzed samples is relatively small (3 normal breast tissues, 6 *in situ* ductal carcinomas, and 11 infiltrating ductal carcinomas) (37). Increasing the number of samples may be necessary to validate this contradictory result. Melanoma cells abnormally express high levels of SOCS3, which influences the responsiveness of melanoma cells to IFN-α and IFN-γ and consequently reduces the efficiency of immunotherapy (49). This study demonstrated that the expression of SOCS3 in melanoma is a disadvantage.

Suppressor of cytokine signaling 3 regulates numerous tumors through inhibition of various signaling pathways and functioning as a tumor suppressor gene. Similar to SOCS1, the expression and function of SOCS3 vary significantly among different tumor types. SOCS3 also promotes the progression of melanoma and attenuates the therapeutic efficacy of IFN-α and IFN-γ treatments. Therefore, a comprehensive understanding of the functions and mechanisms regulating the expression of SOCS1 and SOCS3 might facilitate their future clinical applications, either as diagnostic or prognostic biomarkers.

## SOCS and Immunocytes

### SOCS in DCs

Suppressor of cytokine signaling 1 is critical in the regulation of the function of DCs (11). SOCS1-silenced DCs can induce an enhanced antigen-specific CTL response and antitumor activity (50). SOCS1 can maintain the self-tolerance of DCs at the host level through the constraint of IL-12 and downstream cytokine signaling cascade (51). Immunization with SOCS1^−/−^ DCs induces hyper Th1 immune responses and antitumor activities (52). During DCs differentiation, the STAT pathway is developmentally regulated; considerable increases of SOCS1, SOCS2, and SOCS3 are correlated with the downregulation of STAT6 (53) and regulate DCs maturation. In addition to the regulation of phosphokinase, SOCS3 can also regulate the metabolic enzyme in DCs. Indoleamine 2,3-dioxygenase (IDO) is a rate-limiting enzyme in the catabolic process of tryptophan, which is an essential amino acid for T-cell proliferation and activation (54). IDO^+^ DCs are a subset of human tolerogenic DCs that play a pivotal role in establishing an immunosuppressive microenvironment. The degradation of IDO in CD8^+^ DCs is mediated by SOCS3. SOCS3 binds mouse IDO, recruits the Elongin–Cullin–SOCS E3 ligase, and targets the IDO/SOCS3 complex for proteasomal degradation (55). Moreover, the IL-6-dependent upregulation of SOCS3 is responsible for inhibiting the IFN-driven transcriptional expression of IDO (56). Thus, an inverse relationship exists in DCs between SOCS3 and IDO. Furthermore, SOCS3 can bind to pyruvate kinase type M2 (M2-PK) in DCs. M2-PK is a key enzyme in glycolysis. The interaction of SOCS3 with M2-PK reduces the ATP production and weakens the curative effect of antitumor immune therapy, which indicates that SOCS3 manipulates the activation and function of DCs in the tumor microenvironment (57) (Figure 1A). Hence, depending on the regulated enzymes, SOCS3 is probably both a promoter and an inhibitor for the function of DCs.

### SOCS in Macrophages and MDSCs

Macrophages express SOCS1 and SOCS3 (58, 59), and they are both rapidly inducible and present potent but distinct inhibitory roles. The increased infiltrating numbers of macrophages express SOCS3 in tumors with complete responses compared with those without response to chemotherapy (60). Given that SOCS3 inhibits the STAT3 signaling that is responsible for the inflammatory cytokine section of macrophage and that STAT3 signaling is highly activated in M1 macrophages, the expression of SOCS3 significantly regulates the macrophage differentiation and its proinflammatory properties (61).

Suppressor of cytokine signaling 3 is an important controller of macrophage phases and functions, which could regulate macrophage polarization. In macrophages, SOCS3 is knocked down by siRNA-prevented M1 activation; consequently, STAT3 activity is enhanced and the syntheses of NO and IL-6 is decreased, thereby suggesting that SOCS3 is necessary for development of M1 macrophages (13, 62). Recently, SOCS3 deficiency in macrophages exhibits a remarkable bias toward M2-like population and results in resistance to LPS-induced endotoxic shock. Conversely, SOCS2 deficiency enriches the M1-like phenotype. The SOCS3 in the macrophages suppresses M2 by inhibiting IL-4- and IL-12-induced STAT6 phosphorylation (63). Thus, SOCS3 is an essential controller of macrophage polarization and function, thereby promoting anti-inflammatory responses in the case of SOCS3 deficiency. Nevertheless, some studies proposed conflicting opinions. Some reports demonstrated that SOCS3 deficiency promotes M1 macrophage polarization in tumors (64, 65). In glioma, SOCS3-deficient bone marrow-derived macrophages display enhanced and prolonged expression of proinflammatory M1 cytokines, which contradicts the observation in inflammation (65). Suppression of SOCS3 (specific SOCS3 conditional knockout) in macrophages prevents cancer metastasis by modifying macrophage phase and inducing the production of antitumorigenic chemokine MCP2/CCL8 (61). These results implied that SOCS3 exerts remarkably different effects on macrophage differentiation and promotes M1 polarization in tumor deficiency of SOCS3. Inflammation overexpression of SOCS3 confers similar effects. The difference between these two phenomena might be attributed to the different microenvironments.

The genetic deletion of SOCS3, specifically in myeloid cells, significantly enhances tumor growth, which correlates with elevated levels of MDSCs in the tumor microenvironment. SOCS3 negatively regulates the G-CSF-induced generation of Gr-1^+^CD11b^+^ cells by inhibiting the JAK/STAT3 activation (66). The loss of SOCS3 in myeloid cells promotes the differentiation of BM-derived progenitor cells into Gr-1^+^CD11b^+^ MDSCs and the generation of immunosuppressive microenvironment (66). Their findings clearly demonstrated the importance of SOCS3 in restricting MDSC-mediated immunosuppression activity in tumor (Figures 1B,C). Further work should be performed to verify the critical function of SOCS3 as a negative regulator of MDSCs development and function in tumor environment.

### SOCS in T Cells

The inhibition of Th1 differentiation by IL-6 is mediated by SOCS1. IL-6 upregulates the SOCS1 expression in activated CD4^+^ T cells, thereby interfering with the STAT1 phosphorylation and blocking Th1 differentiation (67). SOCS3 regulates the activation and differentiation of naive CD4^+^ T cells, preferentially promoting Th2 and inhibiting Th1 differentiation through the inhibition of IL-12-mediated STAT4 activation (68). SOCS1 and SOCS3 function contradictory in Th1 differentiation. SOCS1-deficient naive CD4^+^ T cells are predominantly differentiated into Th1 and poorly into Th17 *in vitro*. A large amount of IFN-γ in SOCS1-deficient T cells suppresses Th17 differentiation, which can be explained by the SOCS3-induced STAT3 suppression after STAT1 activation in SOCS1-deficient T cells (69). The TGF-β-mediated SMAD transcriptional activity is severely inhibited in SOCS1-deficient cells in the presence of IFN-γ. Therefore, SOCS1 is necessary for Th17 differentiation by suppressing the antagonistic effect of IFN-γ on both STAT3 and SMAD (69). SOCS1 promotes Th17 differentiation through upregulating STAT3 and SMAD signaling. Moreover, targeting SOCS1 in the T-cell compartment could represent a novel means to enhance the antitumor activity of exogenously administered IFN-α in melanoma (70). The overexpression of SOCS1 and SOCS3 in T cells inhibits IFN-α-induced phosphorylated STAT1 and the transcription of IFN-stimulated genes (3). Unlike the T-helper cells, Tregs are deficient in SOCS3 expression. The *in vitro* overexpression of SOCS3 in Tregs decreases their proliferation and Foxp3 expression. Similarly, some research found that SOCS3 removal in T-lymphocytes upregulates CTLA-4 expression, which shows that SOCS3 negatively regulates CTLA-4 level in T cells and provides a mechanistic explanation for the expansion of Tregs (71). IL-12 permits the conversion of mouse IL-17-producing CD8^+^ T (Tc17) cells to IL-17/IFN-γ-double producing CD8^+^ T (Tc17/IFN-γ) cells by the prohibitive epigenetic modifications of the SOCS3 gene promoters (72). This conversion mechanism for therapeutic tumor immunity can be manipulated because these Tc17/IFN-γ cells exhibit potent antitumor activities (Figure 1D).

## Regulation on SOCS Expression

### Epigenetic and Genetic Dysregulations of SOCS1 and SOCS3

The epigenetic dysregulation of SOCS family genes frequently occurs in cancer and causes gene silence, protein downregulation, and inactivation in relatively large number of tumor cells. The methylation status of SOCS genes varies in different types of tumors (73). SOCS3 hypermethylation is observed in cholangiocarcinoma (74, 75), head and neck squamous cell carcinoma (8), and lung cancer (76). SOCS1 hypermethylation is detected in myeloid leukemia (77–79), lymphoma (80), Barrett’s adenocarcinoma (81), and breast cancer (73). However, SOCS1 hypermethylation shows no correlation with clinical features, OS, and PFS in acute myeloid leukemia patients (79). Both phenomena can be detected in hepatocellular carcinoma (9, 82). miR-29b induces the demethylation of the SOCS1 gene promoter, which results in SOCS1 protein upregulation that is associated with reduced STAT3 phosphorylation and impaired NF-κB activity (83). The acetylation status of SOCS genes also influences the expression and function of SOCS proteins. Histone deacetylase inhibitor, that is, sodium butyrate, increases the transcript and protein expression levels of SOCS1 and SOCS3 by triggering the histone acetylation of SOCS1 and SOCS3 gene promoters in myeloproliferative neoplasm (84). Trichostatin A (TSA), a histone deacetylase inhibitor, suppresses JAK2/STAT3 signaling by increasing the expression of SOCS1 and SOCS3 in human colorectal cancer cells (85) and manipulates the expression of SOCS proteins in tumors. The somatic hypermutation of SOCS1 in lymphocyte-predominant Hodgkin lymphoma (lpHL) is accompanied with high JAK2 expression and STAT6 activation (86). Furthermore, the genomic deletions of the region encompassing SOCS1 are frequent in lpHL and affect the expression of SOCS1 (87). Several genomic single nucleotide polymorphisms within the locus of SOCS1 are associated with the dysfunction of SOCS1 in leukemia (88).

### microRNA (miRNA) Regulation on SOCS1 and SOCS3

microRNAs are small non-coding RNAs that bind to complementary sequences on target mRNAs to induce gene silence and manipulate gene functions (89). miRNA-155 can modulate the differentiation and function of Th17 cells by targeting SOCS1. Pre-miR-155 inhibits the expression of SOCS1 and enhances the phosphorylation of STAT3 and STAT5 proteins (90). miR-19a and miR-19b could downregulate the expression of SOCS1 and SOCS3 in various tumors. miR-19a directly binds the 3′-UTR of the SOCS1 and regulates its expression in NSCLC cells; the transfection with miR-19a mimics significantly decreases the mRNA and protein levels of SOCS1 (91). Similarly, miR-19a significantly decreases SOCS3 mRNA and protein, whereas a miR-19a antagonist specifically reverses the inhibitory effect of miR-19a to SOCS3 (92). Furthermore, the miR-19a-mediated reduction of SOCS3 enhances the IFN-α and IL-6 signal transductions through STAT3 (92). The overexpression of miR-19b in human colon cancer cell line HT-29 cells downregulates the protein level of SOCS3, but not that of the SOCS3 mRNA. The downregulation of SOCS3 by miR-19b causes the phosphorylation of STAT3 and induces the expression of cyclinD1, which could promote cell proliferation by the transition from G1 phase to S and G2 phases (93). miR-221 directly inhibits the expression of SOCS3 and sensitizes prostate cancer cells to IFN-γ-mediated growth inhibition (94). Therefore, although few references have reported that miRNA-induced dysregulation of SOCS expression and function exerts significant influence on the clinical outcome of cancer patients, some miRNAs, such as miR-155, miR-19a, miR-19b, and miR-221, might be promising biomarker candidates in predicting and evaluating the clinical prognosis of certain tumor types.

## SOCS Target Therapy

As the pivotal negative regulators of cytokine signaling both in tumor cells and immunocytes, the dysregulation of the expression and function of SOCS1 and SOCS3 proteins significantly promotes tumor growth, migration, and invasion. Hence, further investigation on the feasibility of SOCS1 and SOCS3 as potential cancer therapeutic targets has been conducted in many types of cancers.

Nevertheless, considering the disparity in the expression patterns of SOCS1 and SOCS3 in tumor cells and immunocytes, contradicting therapeutic approaches have been proposed. Overexpression of SOCS proteins in tumor cells is one approach to inhibit tumor growth by suppressing tumor-promoting STATs. Downregulating the expression of SOCS proteins in immunocytes, such as DCs and T cells, enhances the antitumor immunity by increasing STATs activation (16). The infection of cells with oncolytic adenovirus CN305 (AdCN305)-SOCS3 and AdCN305 cell-penetrating peptides-SOCS3 (membrane permeable SOCS3) results in considerable cytotoxicity of liver tumor cells and downregulation of cyclin D1 and Bcl-xL (7, 95). Tyrosine kinase inhibitor peptide, Tkip, was developed as a mimetic of SOCS proteins and effectively inhibited the JAK2-mediated phosphorylation of STAT1 and proliferation of prostate cancer cells (96, 97). Platelet factor-4 enhances the expression of SOCS3 protein, thereby suppressing the STAT3 activation and inducing cell apoptosis in myeloma (98). TSA increases the expression of SOCS1 and SOCS3 in human colorectal cancer cells and suppresses CRC growth (85). Dehydrocostus lactone, a plant-derived sesquiterpene lactone, inhibits cell proliferation by inducing cell cycle arrest and apoptosis through upregulating SOCS1 and SOCS3 in breast cancer cells (99). The exogenous expression of SOCS3 in MCF-7 cells increases the sensitization to cisplatin-mediated apoptosis, which implied that SOCS target therapeutic strategy may be helpful to overcome cisplatin resistance in breast cancer patients (100). In addition, the transfection of SOCS1 siRNA in DCs significantly enhances the immunogenicity of DC-based tumor vaccines to break self-tolerance and induces effective antitumor immunity (101, 102). Similarly, silencing SOCS1 in macrophages to prompt antitumor immunity also yielded encouraging results (103).

## Concluding Remarks

Following the discovery of the SOCS protein family, the dysregulation of SOCS proteins has been identified in many types of tumor cells and immunocytes through different molecular mechanisms. SOCS proteins are mainly responsible for the dysfunctional negative feedback loops upon cytokine stimulation, which promote oncogenic transformation and tumor cell invasion by inducing the constitutive activation of the STAT signaling pathway. Thus, specific treatment targeting SOCS1 and SOCS3 might be considerably important in inhibiting tumor development and progression. However, considering the difference in the expression patterns of SOCS1 and SOCS3 in tumor cells and immunocytes, different therapeutic approaches should be implemented according to the types of target cells. Downregulating the expression of SOCS proteins in immunocytes might be utilized as a promising strategy because it significantly reverses the immunosuppressive microenvironment and enhances antitumor immune surveillance by enhancing the antitumor immunity of DCs, macrophages, and T-cells in the host. Therefore, further studies on the SOCS family will provide new insights into the comprehensive understanding of the molecular mechanisms of SOCS-mediated tumor progression and the development of immunosuppressive microenvironment. These studies will also help oncologists to determine efficient therapeutic approaches that enhance the antitumor immunity and reduce tumor invasion and metastasis.

## Author Contributions

The authors MJ, JY, W-wZ, PL, WY, and TL declare that there are no conflicts of interest. All authors read and approved the final manuscript.

## Conflict of Interest Statement

The authors declare that the research was conducted in the absence of any commercial or financial relationships that could be construed as a potential conflict of interest.

## References

[1] Babon JJ Varghese LN Nicola NA. Inhibition of IL-6 family cytokines by SOCS3. Semin Immunol (2014) 26(1):13–9. 10.1016/j.smim.2013.12.004 24418198 PMC3970923

[2] Zhao CL Zhang GP Xiao ZZ Ma ZK Lei CP Song SY Recombinant human granulocyte colony-stimulating factor promotes preinvasive and invasive estrogen receptor-positive tumor development in MMTV-erbB2 mice. J Breast Cancer (2015) 18(2):126–33. 10.4048/jbc.2015.18.2.126 26155288 PMC4490261

[3] Zimmerer JM Lesinski GB Kondadasula SV Karpa VI Lehman A RayChaudhury A IFN-alpha-induced signal transduction, gene expression, and antitumor activity of immune effector cells are negatively regulated by suppressor of cytokine signaling proteins. J Immunol (2007) 178(8):4832–45. 10.4049/jimmunol.178.8.4832 17404264

[4] Fojtova M Boudny V Kovarik A Lauerova L Adamkova L Souckova K Development of IFN-gamma resistance is associated with attenuation of SOCS genes induction and constitutive expression of SOCS 3 in melanoma cells. Br J Cancer (2007) 97(2):231–7. 10.1038/sj.bjc.6603849 17579625 PMC2360293

[5] Inagaki-Ohara K Mayuzumi H Kato S Minokoshi Y Otsubo T Kawamura YI Enhancement of leptin receptor signaling by SOCS3 deficiency induces development of gastric tumors in mice. Oncogene (2014) 33(1):74–84. 10.1038/onc.2012.540 23178499

[6] Haura EB Turkson J Jove R. Mechanisms of disease: insights into the emerging role of signal transducers and activators of transcription in cancer. Nat Clin Pract Oncol (2005) 2(6):315–24. 10.1038/ncponc0195 16264989

[7] Inagaki-Ohara K Kondo T Ito M Yoshimura A. SOCS, inflammation, and cancer. JAKSTAT (2013) 2(3):e24053. 10.4161/jkst.24053 24069550 PMC3772102

[8] Weber A Hengge UR Bardenheuer W Tischoff I Sommerer F Markwarth A SOCS-3 is frequently methylated in head and neck squamous cell carcinoma and its precursor lesions and causes growth inhibition. Oncogene (2005) 24(44):6699–708. 10.1038/sj.onc.1208818 16007169

[9] Niwa Y Kanda H Shikauchi Y Saiura A Matsubara K Kitagawa T Methylation silencing of SOCS-3 promotes cell growth and migration by enhancing JAK/STAT and FAK signalings in human hepatocellular carcinoma. Oncogene (2005) 24(42):6406–17. 10.1038/sj.onc.1208788 16007195

[10] Culig Z. Suppressors of cytokine signalling-3 and -1 in human carcinogenesis. Front Biosci (Schol Ed) (2013) 5:277–83. 10.2741/S372 23277051

[11] Rabinovich GA Gabrilovich D Sotomayor EM. Immunosuppressive strategies that are mediated by tumor cells. Annu Rev Immunol (2007) 25:267–96. 10.1146/annurev.immunol.25.022106.141609 17134371 PMC2895922

[12] Shen L Evel-Kabler K Strube R Chen SY. Silencing of SOCS1 enhances antigen presentation by dendritic cells and antigen-specific anti-tumor immunity. Nat Biotechnol (2004) 22(12):1546–53. 10.1038/nbt1035 15558048

[13] Yoshimura A Suzuki M Sakaguchi R Hanada T Yasukawa H SOCS, inflammation, and autoimmunity. Front Immunol (2012) 3:20 10.3389/fimmu.2012.00020 22566904 PMC3342034

[14] Liongue C Ward AC. Evolution of the JAK-STAT pathway. JAKSTAT (2013) 2(1):e22756. 10.4161/jkst.22756 24058787 PMC3670263

[15] Liongue C O’Sullivan LA Trengove MC Ward AC. Evolution of JAK-STAT pathway components: mechanisms and role in immune system development. PLoS One (2012) 7(3):e32777. 10.1371/journal.pone.0032777 22412924 PMC3296744

[16] Zhang J Li H Yu JP Wang SE Ren XB. Role of SOCS1 in tumor progression and therapeutic application. Int J Cancer (2012) 130(9):1971–80. 10.1002/ijc.27318 22025331

[17] Linossi EM Chandrashekaran IR Kolesnik TB Murphy JM Webb AI Willson TA Suppressor of cytokine signaling (SOCS) 5 utilises distinct domains for regulation of JAK1 and interaction with the adaptor protein Shc-1. PLoS One (2013) 8(8):e70536. 10.1371/journal.pone.0070536 23990909 PMC3749136

[18] Kario E Marmor MD Adamsky K Citri A Amit I Amariglio N Suppressors of cytokine signaling 4 and 5 regulate epidermal growth factor receptor signaling. J Biol Chem (2005) 280(8):7038–48. 10.1074/jbc.M408575200 15590694

[19] Starr R Willson TA Viney EM Murray LJ Rayner JR Jenkins BJ A family of cytokine-inducible inhibitors of signalling. Nature (1997) 387(6636):917–21. 10.1038/43206 9202125

[20] Endo TA Masuhara M Yokouchi M Suzuki R Sakamoto H Mitsui K A new protein containing an SH2 domain that inhibits JAK kinases. Nature (1997) 387(6636):921–4. 10.1038/43213 9202126

[21] Valentino L Pierre J. JAK/STAT signal transduction: regulators and implication in hematological malignancies. Biochem Pharmacol (2006) 71(6):713–21. 10.1016/j.bcp.2005.12.017 16426581

[22] Narazaki M Fujimoto M Matsumoto T Morita Y Saito H Kajita T Three distinct domains of SSI-1/SOCS-1/JAB protein are required for its suppression of interleukin 6 signaling. Proc Natl Acad Sci U S A (1998) 95(22):13130–4. 10.1073/pnas.95.22.13130 9789053 PMC23734

[23] Yasukawa H Misawa H Sakamoto H Masuhara M Sasaki A Wakioka T The JAK-binding protein JAB inhibits Janus tyrosine kinase activity through binding in the activation loop. EMBO J (1999) 18(5):1309–20. 10.1093/emboj/18.5.1309 10064597 PMC1171221

[24] Croker BA Kiu H Nicholson SE. SOCS regulation of the JAK/STAT signalling pathway. Semin Cell Dev Biol (2008) 19(4):414–22. 10.1016/j.semcdb.2008.07.010 18708154 PMC2597703

[25] Babon JJ Kershaw NJ Murphy JM Varghese LN Laktyushin A Young SN Suppression of cytokine signalling by SOCS3: characterisation of the mode of inhibition and the basis of its specificity. Immunity (2012) 36(2):239–50. 10.1016/j.immuni.2011.12.015 22342841 PMC3299805

[26] Kazi JU Kabir NN Flores-Morales A Rönnstrand L. SOCS proteins in regulation of receptor tyrosine kinase signaling. Cell Mol Life Sci (2014) 71(17):3297–310. 10.1007/s00018-014-1619-y 24705897 PMC11113172

[27] Chan SR Rickert CG Vermi W Sheehan KC Arthur C Allen JA Dysregulated STAT1-SOCS1 control of JAK2 promotes mammary luminal progenitor cell survival and drives ERα(+) tumorigenesis. Cell Death Differ (2014) 21(2):234–46. 10.1038/cdd.2013.116 24037089 PMC3890946

[28] Iglesias-Gato D Chuan YC Wikström P Augsten S Jiang N Niu Y SOCS2 mediates the cross talk between androgen and growth hormone signaling in prostate cancer. Carcinogenesis (2014) 35(1):24–33. 10.1093/carcin/bgt304 24031028

[29] Lang R Pauleau AL Parganas E Takahashi Y Mages J Ihle JN SOCS3 regulates the plasticity of gp130 signaling. Nat Immunol (2003) 4(6):546–50. 10.1038/ni932 12754506

[30] Yamamoto K Yamaguchi M Miyasaka N Miura O SOCS-3 inhibits IL-12-induced STAT4 activation by binding through its SH2 domain to the STAT4 docking site in the IL-12 receptor β2 subunit. Biochem Biophys Res Commun (2003) 310(4):1188–93. 10.1016/j.bbrc.2003.09.140 14559241

[31] Souma Y Nishida T Serada S Iwahori K Takahashi T Fujimoto M Antiproliferative effect of SOCS-1 through the suppression of STAT3 and p38 MAPK activation in gastric cancer cells. Int J Cancer (2012) 131(6):1287–96. 10.1002/ijc.27350 22095154

[32] Liu E Côté J-F Vuori K. Negative regulation of FAK signaling by SOCS proteins. EMBO J (2003) 22(19):5036–46. 10.1093/emboj/cdg503 14517242 PMC204486

[33] Shimada K Serada S Fujimoto M Nomura S Nakatsuka R Harada E Molecular mechanism underlying the antiproliferative effect of suppressor of cytokine signaling-1 in non-small-cell lung cancer cells. Cancer Sci (2013) 104(11):1483–91. 10.1111/cas.12266 23962256 PMC7654259

[34] Neuwirt H Puhr M Santer FR Susani M Doppler W Marcias G Suppressor of cytokine signaling (SOCS)-1 is expressed in human prostate cancer and exerts growth-inhibitory function through down-regulation of cyclins and cyclin-dependent kinases. Am J Pathol (2009) 174(5):1921–30. 10.2353/ajpath.2009.080751 19342366 PMC2671279

[35] David M Naudin C Letourneur M Polrot M Renoir JM Lazar V Suppressor of cytokine signaling 1 modulates invasion and metastatic potential of colorectal cancer cells. Mol Oncol (2014) 8(5):942–55. 10.1016/j.molonc.2014.03.014 24726456 PMC5528518

[36] Evans MK Yu CR Lohani A Mahdi RM Liu X Trzeciak AR Expression of SOCS1 and SOCS3 genes is differentially regulated in breast cancer cells in response to proinflammatory cytokine and growth factor signals. Oncogene (2007) 26(13):1941–8. 10.1038/sj.onc.1209993 17001312

[37] Raccurt M Tam SP Lau P Mertani HC Lambert A Garcia-Caballero T Suppressor of cytokine signalling gene expression is elevated in breast carcinoma. Br J Cancer (2003) 89(3):524–32. 10.1038/sj.bjc.6601115 12888825 PMC2394374

[38] Tam SP Lau P Djiane J Hilton DJ Waters MJ. Tissue-specific induction of SOCS gene expression by PRL. Endocrinology (2001) 142(11):5015–26. 10.1210/endo.142.11.8466 11606470

[39] Smolkova B Mego M Horvathova Kajabova V Cierna Z Danihel L Sedlackova T Expression of SOCS1 and CXCL12 proteins in primary breast cancer are associated with presence of circulating tumor cells in peripheral blood. Transl Oncol (2016) 9(3):184–90. 10.1016/j.tranon.2016.03.004 27267835 PMC4856862

[40] Sasi W Jiang WG Sharma A Mokbel K. Higher expression levels of SOCS 1,3,4,7 are associated with earlier tumour stage and better clinical outcome in human breast cancer. BMC Cancer (2010) 10:178. 10.1186/1471-2407-10-178 20433750 PMC2876081

[41] Roman-Gomez J Jimenez-Velasco A Castillejo JA Cervantes F Barrios M Colomer D The suppressor of cytokine signaling-1 is constitutively expressed in chronic myeloid leukemia and correlates with poor cytogenetic response to interferon-alpha. Haematologica (2004) 89(1):42. 14754605

[42] Alexander WS Starr R Fenner JE Scott CL Handman E Sprigg NS SOCS1 is a critical inhibitor of interferon γ signaling and prevents the potentially fatal neonatal actions of this cytokine. Cell (1999) 98(5):597–608. 10.1016/S0092-8674(00)80047-1 10490099

[43] Starr R Metcalf D Elefanty AG Brysha M Willson TA Nicola NA Liver degeneration and lymphoid deficiencies in mice lacking suppressor of cytokine signaling-1. Proc Natl Acad Sci U S A (1998) 95(24):14395–9. 10.1073/pnas.95.24.14395 9826711 PMC24384

[44] Neuwirt H Puhr M Cavarretta IT Mitterberger M Hobisch A Culig Z. Suppressor of cytokine signalling-3 is up-regulated by androgen in prostate cancer cell lines and inhibits androgen-mediated proliferation and secretion. Endocr Relat Cancer (2007) 14(4):1007–19. 10.1677/ERC-07-0172 18045952

[45] Iwahori K Serada S Fujimoto M Nomura S Osaki T Lee CM Overexpression of SOCS3 exhibits preclinical antitumor activity against malignant pleural mesothelioma. Int J Cancer (2011) 129(4):993–1005. 10.1002/ijc.25716 20949562

[46] Lehmann U Schmitz J Weissenbach M Sobota RM Hortner M Friederichs K SHP2 and SOCS3 contribute to Tyr-759-dependent attenuation of interleukin-6 signaling through gp130. J Biol Chem (2003) 278(1):661–71. 10.1074/jbc.M210552200 12403768

[47] Kim G Ouzounova M Quraishi AA Davis A Tawakkol N Clouthier SG SOCS3-mediated regulation of inflammatory cytokines in PTEN and p53 inactivated triple negative breast cancer model. Oncogene (2015) 34(6):671–80. 10.1038/onc.2014.4 24531711 PMC4285772

[48] Li Y Deuring J Peppelenbosch MP Kuipers EJ de Haar C van der Woude CJ. IL-6-induced DNMT1 activity mediates SOCS3 promoter hypermethylation in ulcerative colitis-related colorectal cancer. Carcinogenesis (2012) 33(10):1889–96. 10.1093/carcin/bgs214 22739025

[49] Lesinski GB Zimmerer JM Kreiner M Trefry J Bill MA Young GS Modulation of SOCS protein expression influences the interferon responsiveness of human melanoma cells. BMC Cancer (2010) 10:142. 10.1186/1471-2407-10-142 20398276 PMC2858748

[50] Shen L. Silencing of SOCS1 enhances antigen presentation by dendritic cells and antigen-specific anti-tumor immunity. Nat Biotechnol (2004) 22(12):1546–53. 10.1038/nbt1035 15558048

[51] Evel-Kabler K Song XT Aldrich M Huang XF Chen SY. SOCS1 restricts dendritic cells’ ability to break self tolerance and induce antitumor immunity by regulating IL-12 production and signaling. J Clin Invest (2006) 116(1):90–100. 10.1172/JCI26169 16357940 PMC1312019

[52] Hanada T Tanaka K Matsumura Y Yamauchi M Nishinakamura H Aburatani H Induction of hyper Th1 cell-type immune responses by dendritic cells lacking the suppressor of cytokine signaling-1 gene. J Immunol (2005) 174(7):4325–32. 10.4049/jimmunol.174.7.4325 15778397

[53] Jackson SH Yu CR Mahdi RM Ebong S Egwuagu CE. Dendritic cell maturation requires STAT1 and is under feedback regulation by suppressors of cytokine signaling. J Immunol (2004) 172(4):2307–15. 10.4049/jimmunol.172.4.2307 14764699

[54] Yu J Sun J Wang SE Li H Cao S Cong Y Upregulated expression of indoleamine 2, 3-dioxygenase in primary breast cancer correlates with increase of infiltrated regulatory T cells in situ and lymph node metastasis. Clin Dev Immunol (2011) 2011:469135. 10.1155/2011/469135 22110525 PMC3202140

[55] Pallotta MT Orabona C Volpi C Grohmann U Puccetti P Fallarino F Proteasomal degradation of indoleamine 2,3-dioxygenase in CD8(+) dendritic cells is mediated by suppressor of cytokine signaling 3 (SOCS3). Int J Tryptophan Res (2010) 3:91–7. 10.4137/IJTR.S3971 22084591 PMC3195250

[56] Fallarino F Orabona C Vacca C Bianchi R Gizzi S Asselin-Paturel C Ligand and cytokine dependence of the immunosuppressive pathway of tryptophan catabolism in plasmacytoid dendritic cells. Int Immunol (2005) 17(11):1429–38. 10.1093/intimm/dxh321 16172135

[57] Zhang Z Liu Q Che Y Yuan X Dai L Zeng B Antigen presentation by dendritic cells in tumors is disrupted by altered metabolism that involves pyruvate kinase M2 and its interaction with SOCS3. Cancer Res (2010) 70(1):89–98. 10.1158/0008-5472.CAN-09-2970 19996282

[58] Yoshimura A Naka T Kubo M. SOCS proteins, cytokine signalling and immune regulation. Nat Rev Immunol (2007) 7(6):454–65. 10.1038/nri2093 17525754

[59] Alexander WS Hilton DJ. The role of suppressors of cytokine signaling (SOCS) proteins in regulation of the immune response. Annu Rev Immunol (2004) 22:503–29. 10.1146/annurev.immunol.22.091003.090312 15032587

[60] Heys SD Stewart KN McKenzie EJ Miller ID Wong SY Sellar G Characterisation of tumour-infiltrating macrophages: impact on response and survival in patients receiving primary chemotherapy for breast cancer. Breast Cancer Res Treat (2012) 135(2):539–48. 10.1007/s10549-012-2190-6 22886449

[61] Hiwatashi K Tamiya T Hasegawa E Fukaya T Hashimoto M Kakoi K Suppression of SOCS3 in macrophages prevents cancer metastasis by modifying macrophage phase and MCP2/CCL8 induction. Cancer Lett (2011) 308(2):172–80. 10.1016/j.canlet.2011.04.024 21624767

[62] Liu Y Stewart KN Bishop E Marek CJ Kluth DC Rees AJ Unique expression of suppressor of cytokine signaling 3 is essential for classical macrophage activation in rodents in vitro and in vivo. J Immunol (2008) 180(9):6270–8. 10.4049/jimmunol.180.9.6270 18424750

[63] Spence S Fitzsimons A Boyd CR Kessler J Fitzgerald D Elliott J Suppressors of cytokine signaling 2 and 3 diametrically control macrophage polarization. Immunity (2013) 38(1):66–78. 10.1016/j.immuni.2012.09.013 23177319

[64] Qin H Holdbrooks AT Liu Y Reynolds SL Yanagisawa LL Benveniste EN Correction: SOCS3 deficiency promotes M1 macrophage polarization and inflammation. J Immunol (2016) 197(1):387–9. 10.4049/jimmunol.1600710 27317734

[65] McFarland BC Marks MP Rowse AL Fehling SC Gerigk M Qin H Loss of SOCS3 in myeloid cells prolongs survival in a syngeneic model of glioma. Oncotarget (2016) 7(15):20621–35. 10.18632/oncotarget.7992 26967393 PMC4991480

[66] Yu H Liu Y McFarland BC Deshane JS Hurst DR Ponnazhagan S SOCS3 deficiency in myeloid cells promotes tumor development: involvement of STAT3 activation and myeloid-derived suppressor cells. Cancer Immunol Res (2015) 3(7):727–40. 10.1158/2326-6066.CIR-15-0004 25649351 PMC4570503

[67] Diehl S Anguita J Hoffmeyer A Zapton T Ihle JN Fikrig E Inhibition of Th1 differentiation by IL-6 is mediated by SOCS1. Immunity (2000) 13(6):805–15. 10.1016/S1074-7613(00)00078-9 11163196

[68] Egwuagu CE Yu CR Zhang M Mahdi RM Kim SJ Gery I. Suppressors of cytokine signaling proteins are differentially expressed in Th1 and Th2 cells: implications for Th cell lineage commitment and maintenance. J Immunol (2002) 168(7):3181–7. 10.4049/jimmunol.168.7.3181 11907070

[69] Tanaka K Ichiyama K Hashimoto M Yoshida H Takimoto T Takaesu G Loss of suppressor of cytokine signaling 1 in helper T cells leads to defective Th17 differentiation by enhancing antagonistic effects of IFN on STAT3 and Smads. J Immunol (2008) 180(6):3746–56. 10.4049/jimmunol.180.6.3746 18322180

[70] Guenterberg KD Lesinski GB Mundy-Bosse BL Karpa VI Jaime-Ramirez AC Wei L Enhanced anti-tumor activity of interferon-alpha in SOCS1-deficient mice is mediated by CD4(+) and CD8(+) T cells. Cancer Immunol Immunother (2011) 60(9):1281–8. 10.1007/s00262-011-1034-2 21604070 PMC3521522

[71] Yu CR Kim SH Mahdi RM Egwuagu CE. SOCS3 deletion in T lymphocytes suppresses development of chronic ocular inflammation via upregulation of CTLA-4 and expansion of regulatory T cells. J Immunol (2013) 191(10):5036–43. 10.4049/jimmunol.1301132 24101549 PMC4041279

[72] Satoh T Tajima M Wakita D Kitamura H Nishimura T The development of IL-17/IFN-gamma-double producing CTLs from Tc17 cells is driven by epigenetic suppression of Socs3 gene promoter. Eur J Immunol (2012) 42(9):2329–42. 10.1002/eji.201142240 22674086

[73] Sutherland KD Lindeman GJ Choong DY Wittlin S Brentzell L Phillips W Differential hypermethylation of SOCS genes in ovarian and breast carcinomas. Oncogene (2004) 23(46):7726–33. 10.1038/sj.onc.1207787 15361843

[74] Isomoto H. Epigenetic alterations in cholangiocarcinoma-sustained IL-6/STAT3 signaling in cholangio-carcinoma due to SOCS3 epigenetic silencing. Digestion (2009) 79(Suppl 1):2–8. 10.1159/000167859 19153483

[75] Isomoto H Mott JL Kobayashi S Werneburg NW Bronk SF Haan S Sustained IL-6/STAT-3 signaling in cholangiocarcinoma cells due to SOCS-3 epigenetic silencing. Gastroenterology (2007) 132(1):384–96. 10.1053/j.gastro.2006.10.037 17241887 PMC2203612

[76] He B You L Uematsu K Zang K Xu Z Lee AY SOCS-3 is frequently silenced by hypermethylation and suppresses cell growth in human lung cancer. Proc Natl Acad Sci U S A (2003) 100(24):14133–8. 10.1073/pnas.2232790100 14617776 PMC283558

[77] Hatirnaz O Ure U Ar C Akyerli C Soysal T Ferhanoğlu B The SOCS-1 gene methylation in chronic myeloid leukemia patients. Am J Hematol (2007) 82(8):729–30. 10.1002/ajh.20886 17315216

[78] Watanabe D Ezoe S Fujimoto M Kimura A Saito Y Nagai H Suppressor of cytokine signalling-1 gene silencing in acute myeloid leukaemia and human haematopoietic cell lines. Br J Haematol (2004) 126(5):726–35. 10.1111/j.1365-2141.2004.05107.x 15327527

[79] Chen CY Tsay W Tang JL Shen HL Lin SW Huang SY SOCS1 methylation in patients with newly diagnosed acute myeloid leukemia. Genes Chromosomes Cancer (2003) 37(3):300–5. 10.1002/gcc.10222 12759928

[80] Weniger MA Melzner I Menz CK Wegener S Bucur AJ Dorsch K Mutations of the tumor suppressor gene SOCS-1 in classical Hodgkin lymphoma are frequent and associated with nuclear phospho-STAT5 accumulation. Oncogene (2006) 25(18):2679–84. 10.1038/sj.onc.1209151 16532038

[81] Tischoff I Hengge UR Vieth M Ell C Stolte M Weber A Methylation of SOCS-3 and SOCS-1 in the carcinogenesis of Barrett’s adenocarcinoma. Gut (2007) 56(8):1047–53. 10.1136/gut.2006.111633 17376806 PMC1955493

[82] Yang B Guo M Herman JG Clark DP. Aberrant promoter methylation profiles of tumor suppressor genes in hepatocellular carcinoma. Am J Pathol (2003) 163(3):1101–7. 10.1016/S0002-9440(10)63469-4 12937151 PMC1868243

[83] Amodio N Bellizzi D Leotta M Raimondi L Biamonte L D’Aquila P miR-29b induces SOCS-1 expression by promoter demethylation and negatively regulates migration of multiple myeloma and endothelial cells. Cell Cycle (2013) 12(23):3650–62. 10.4161/cc.26585 24091729 PMC3903716

[84] Gao SM Chen CQ Wang LY Hong LL Wu JB Dong PH Histone deacetylases inhibitor sodium butyrate inhibits JAK2/STAT signaling through upregulation of SOCS1 and SOCS3 mediated by HDAC8 inhibition in myeloproliferative neoplasms. Exp Hematol (2013) 41(3):261.e–70.e. 10.1016/j.exphem.2012.10.012 23111066

[85] Xiong H Du W Zhang YJ Hong J Su WY Tang JT Trichostatin A, a histone deacetylase inhibitor, suppresses JAK2/STAT3 signaling via inducing the promoter-associated histone acetylation of SOCS1 and SOCS3 in human colorectal cancer cells. Mol Carcinog (2012) 51(2):174–84. 10.1002/mc.20777 21520296

[86] Mottok A Renné C Willenbrock K Hansmann ML Bräuninger A. Somatic hypermutation of SOCS1 in lymphocyte-predominant Hodgkin lymphoma is accompanied by high JAK2 expression and activation of STAT6. Blood (2007) 110(9):3387–90. 10.1182/blood-2007-03-082511 17652621

[87] Franke S Wlodarska I Maes B Vandenberghe P Delabie J Hagemeijer A Lymphocyte predominance Hodgkin disease is characterized by recurrent genomic imbalances. Blood (2001) 97(6):1845. 10.1182/blood.V97.6.1845 11238128

[88] Guillem V Amat P Cervantes F Alvarez-Larrán A Cervera J Maffioli M Functional polymorphisms in SOCS1 and PTPN22 genes correlate with the response to imatinib treatment in newly diagnosed chronic-phase chronic myeloid leukemia. Leuk Res (2012) 36(2):174–81. 10.1016/j.leukres.2011.06.011 21724255

[89] Bartel DP. microRNAs: genomics, biogenesis, mechanism, and function. Cell (2004) 116(2):281–97. 10.1016/S0092-8674(04)00045-5 14744438

[90] Yao R Ma YL Liang W Li HH Ma ZJ Yu X microRNA-155 modulates Treg and Th17 cells differentiation and Th17 cell function by targeting SOCS1. PLoS One (2012) 7(10):e46082. 10.1371/journal.pone.0046082 23091595 PMC3473054

[91] Zhe WXC. microRNA-19a functions as an oncogenic microRNA in non-small cell lung cancer by targeting the suppressor of cytokine signaling 1 and mediating STAT3 activation. Int J Mol Med (2015) 35(3):839–46. 10.3892/ijmm.2015.2071 25604748

[92] Collins AS McCoy CE Lloyd AT O’Farrelly C Stevenson NJ. miR-19a: an effective regulator of SOCS3 and enhancer of JAK-STAT signalling. PLoS One (2013) 8(7):e69090. 10.1371/journal.pone.0069090 23894411 PMC3718810

[93] Cheng X-Q miRNA-19b regulates proliferation of human colon cancer cell line HT-29. Shi Jie Hua Ren Xiao Hua Za Zhi (2015) 23(10):1568 10.11569/wcjd.v23.i10.1568

[94] Kneitz B Krebs M Kalogirou C Schubert M Joniau S van Poppel H Survival in patients with high-risk prostate cancer is predicted by miR-221, which regulates proliferation, apoptosis, and invasion of prostate cancer cells by inhibiting IRF2 and SOCS3. Cancer Res (2014) 74(9):2591–603. 10.1158/0008-5472.CAN-13-1606 24607843

[95] Cui Q Jiang W Wang Y Lv C Luo J Zhang W Transfer of suppressor of cytokine signaling 3 by an oncolytic adenovirus induces potential antitumor activities in hepatocellular carcinoma. Hepatology (2008) 47(1):105–12. 10.1002/hep.21951 17935223

[96] Flowers LO Subramaniam PS Johnson HM. A SOCS-1 peptide mimetic inhibits both constitutive and IL-6 induced activation of STAT3 in prostate cancer cells. Oncogene (2005) 24(12):2114–20. 10.1038/sj.onc.1208437 15688010

[97] Flowers LO Johnson HM Mujtaba MG Ellis MR Haider SM Subramaniam PS. Characterization of a peptide inhibitor of Janus kinase 2 that mimics suppressor of cytokine signaling 1 function. J Immunol (2004) 172(12):7510–8. 10.4049/jimmunol.172.12.7510 15187130

[98] Liang P Cheng SH Cheng CK Lau KM Lin SY Chow EY Platelet factor 4 induces cell apoptosis by inhibition of STAT3 via up-regulation of SOCS3 expression in multiple myeloma. Haematologica (2013) 98(2):288–95. 10.3324/haematol.2012.065607 22929979 PMC3561438

[99] Kuo PL Ni WC Tsai EM Hsu YL. Dehydrocostuslactone disrupts signal transducers and activators of transcription 3 through up-regulation of suppressor of cytokine signaling in breast cancer cells. Mol Cancer Ther (2009) 8(5):1328–39. 10.1158/1535-7163.MCT-08-0914 19383849

[100] Ru P Steele R Hsueh EC Ray RB. Anti-miR-203 upregulates SOCS3 expression in breast cancer cells and enhances cisplatin chemosensitivity. Genes Cancer (2011) 2(7):720–7. 10.1177/1947601911425832 22207897 PMC3218409

[101] Hashimoto M Ayada T Kinjyo I Hiwatashi K Yoshida H Okada Y Silencing ofSOCS1in macrophages suppresses tumor development by enhancing antitumor inflammation. Cancer Sci (2009) 100(4):730–6. 10.1111/j.1349-7006.2009.01098.x 19469017 PMC11159406

[102] Hu Q Qin X Qian G Jiang S Li H Jiang M SOCS1 silencing can break high-dose dendritic cell immunotherapy-induced immune tolerance. Mol Med Rep (2008) 1(1):61–70. 21479379

[103] Song S Wang Y Wang J Lian W Liu S Zhang Z Tumour-derived IL-10 within tumour microenvironment represses the antitumour immunity of Socs1-silenced and sustained antigen expressing DCs. Eur J Cancer (2012) 48(14):2252–9. 10.1016/j.ejca.2011.12.009 22230748

